# HLA-associated outcomes in peanut oral immunotherapy trials identify mechanistic and clinical determinants of therapeutic success

**DOI:** 10.3389/fimmu.2022.941839

**Published:** 2022-11-18

**Authors:** Kanika Kanchan, Gautam Shankar, Michelle F. Huffaker, Henry T. Bahnson, R Sharon Chinthrajah, Srinath Sanda, Monali Manohar, Hua Ling, Justin E. Paschall, George Du Toit, Ingo Ruczinski, Alkis Togias, Gideon Lack, Kari C. Nadeau, Stacie M. Jones, Gerald T. Nepom, Rasika A. Mathias

**Affiliations:** ^1^ Division of Allergy and Clinical Immunology, Department of Medicine, School of Medicine, Johns Hopkins University, Baltimore, MD, United States; ^2^ The Immune Tolerance Network, San Francisco, CA, United States; ^3^ The Immune Tolerance Network, Seattle, WA, United States; ^4^ Benaroya Research Institute at Virginia Mason, Seattle, WA, United States; ^5^ Sean N. Parker Center for Allergy and Asthma Research, Stanford University, Palo Alto, CA, United States; ^6^ Institute of Genetic Medicine, Department of Medicine, School of Medicine, Johns Hopkins University, Baltimore, MD, United States; ^7^ The Department of Pediatric Allergy, Division of Asthma, Allergy and Lung Biology, King’s College London, and Guy’s and St Thomas’ National Health Service (NHS) Foundation Trust, London, United Kingdom; ^8^ Department of Biostatistics, Bloomberg School of Public Health, Johns Hopkins University, Baltimore, MD, United States; ^9^ Division of Allergy, Immunology, and Transplantation, National Institute of Allergy and Infectious Diseases, National Institutes of Health, Rockville, MD, United States; ^10^ Department of Pediatrics, University of Arkansas for Medical Sciences and Arkansas Children’s Hospital, Little Rock, AR, United States

**Keywords:** peanut allergy, oral immunotherapy, HLA, desensitization, remission, tolerance

## Abstract

**Rationale:**

Previous studies identified an interaction between HLA and oral peanut exposure. HLA-DQA1*01:02 had a protective role with the induction of Ara h 2 epitope-specific IgG4 associated with peanut consumption during the LEAP clinical trial for prevention of peanut allergy, while it was a risk allele for peanut allergy in the peanut avoidance group. We have now evaluated this gene-environment interaction in two subsequent peanut oral immunotherapy (OIT) trials - IMPACT and POISED - to better understand the potential for the HLA-DQA1*01:02 allele as an indicator of higher likelihood of desensitization, sustained unresponsiveness, and peanut allergy remission.

**Methods:**

We determined HLA-DQA1*01:02 carrier status using genome sequencing from POISED (N=118, age: 7-55yr) and IMPACT (N=126, age: 12-<48mo). We tested for association with remission, sustained unresponsiveness (SU), and desensitization in the OIT groups, as well as peanut component specific IgG4 (psIgG4) using generalized linear models and adjusting for relevant covariates and ancestry.

**Results:**

While not quite statistically significant, a higher proportion of HLA-DQA1*01:02 carriers receiving OIT in IMPACT were desensitized (93%) compared to non-carriers (78%); odds ratio (OR)=5.74 (p=0.06). In this sample we also observed that a higher proportion of carriers achieved remission (35%) compared to non-carriers (22%); OR=1.26 (p=0.80). In POISED, carriers more frequently attained continued desensitization (80% versus 61% among non-carriers; OR=1.28, p=0.86) and achieved SU (52% versus 31%; OR=2.32, p=0.19). psIgG4 associations with HLA-DQA1*01:02 in the OIT arm of IMPACT which included younger study subjects recapitulated patterns noted in LEAP, but no associations of note were observed in the older POISED study subjects.

**Conclusions:**

Findings across three clinical trials show a pattern of a gene environment interaction between HLA and oral peanut exposure. Age, and prior sensitization contribute additional determinants of outcomes, consistent with a mechanism of restricted antigen recognition fundamental to driving protective immune responses to OIT.

## Introduction

The Learning Early About Peanut Allergy (LEAP) study (1) established that the early dietary introduction of peanut consumption significantly decreased the prevalence of peanut allergy (PA) and modulated immune responses to peanuts among high-risk children. The LEAP participants were randomized into two groups: a consumption group with continuous consumption of peanut, and an avoidance group. The LEAP clinical trial setting offers a unique opportunity to identify genetic determinants of PA risk and of immunological markers of response to peanut exposure in the context of gene environment interactions (2, 3). In the LEAP study, we recently found strong evidence for a gene environment interaction between HLA-DQA1*01:02 and consumption of peanut determining elevated peanut specific IgG4 (psIgG4), particularly for IgG4 to Ara h 2. Notably, we also observed that the same HLA allele increased risk to PA in the absence of peanut exposure (3). This latter finding is consistent with prior genetic studies of PA that did not specifically model peanut exposure; HLA-DQA1*01:02 is a reported risk allele for PA and has not been found to be a risk allele for other food allergens (4). Here, we are investigating these associations in two peanut oral immunotherapy (PnOIT) trials: Oral Immunotherapy for Induction of Tolerance and Desensitization in Peanut-Allergic Children (IMPACT) and The Peanut Oral Immunotherapy Study: Safety, Efficacy and Discovery (POISED).

The IMPACT study is a randomized, double-blind, placebo-controlled trial of peanut PnOIT in peanut allergic children ages 12-<48 months (5). The primary endpoint was desensitization (tolerating a predetermined dose of peanut delivered *via* oral food challenge while still receiving PnOIT) after 134 weeks of treatment and a secondary endpoint was remission (still tolerating that dose of peanut) 26 weeks after treatment discontinuation. The key findings of IMPACT are: (i) 71% participants from the PnOIT treated group compared to 2% from the placebo treated group were desensitized at week 134, and (ii) 21% participants on PnOIT compared to 2.0% on placebo met remission criteria after 26 weeks of OIT avoidance. An important observation was that younger participants (<24 months) had higher probability of remaining protected from peanut allergic reactions after PnOIT discontinuation, compared to those of older age suggesting that early intervention presents a window of opportunity to induce remission against PA in peanut allergic children.

POISED is a single-site, double-blind, randomized, long-term trial of PnOIT in peanut allergic children and adults ages 7-55 years (6). This trial aimed to evaluate: (i) whether PnOIT for 104 weeks followed by 12 weeks of avoidance induces sustained protection from peanut allergic reactions at week 117 (described as sustained unresponsiveness [SU]); (ii) whether a reduced dose (300 mg) of PnOIT given after 104 weeks on the initial higher dose (4000 mg) could maintain protection against peanut at week 117 compared to total PnOIT discontinuation. The main results of the trial are: (i) 84% of the total active arm participants were protected against a peanut oral challenge at week 104, as opposed to 4% of those receiving placebo, (ii) 35% of participants in the peanut discontinuation group remained protected at week 117, as compared to 4% from the placebo group, and (iii) the percentage of participants remaining protected at week 117 in the treatment discontinuation and the treatment reduction groups was 35% and 54%, respectively. Immunological markers, psIgG4 and IgG4 to Ara h 2, were higher in the PnOIT groups compared to placebo. This study supported prior findings that PnOIT discontinuation leads to loss of protection to 4000 mg in a substantial percentage of PnOIT recipients and added the observation that the loss of protection to 4000 mg can occur even with reduction to a lower PnOIT dose, albeit in a smaller percentage of recipients.

In this report, we specifically evaluated if the HLA-DQA1*01:02 associations with psIgG4 responses to peanut consumption in LEAP were also seen with PnOIT in the IMPACT and POISED studies and whether HLA-DQA1*01:02 was also associated with better clinical outcomes in these studies. All three clinical trials reported higher levels of psIgG4 in their respective intervention arms. The interest in IgG4 stems from its potential role as a blocking antibody in antigen immunotherapy (7, 8). We were able to replicate the associations of HLA-DQA1*01:02 with elevated psIgG4 and Ara h 2 IgG4 identified in the LEAP consumption group in the PnOIT group of the IMPACT study but not in the POISED study, and we observed that a higher proportion of HLA-DQA1*01:02 allele carriers in both IMPACT and POISED showed desensitization, remission, and continued protection after treatment discontinuation or treatment dose reduction, as compared to non-carriers of the allele.

## Materials and methods

### Study designs of the OIT trials

Study design for both OIT trials, IMPACT (5) and POISED (6) were published previously and shown in **Figure S1**. Briefly, there are two treatment groups: PnOIT and placebo in the IMPACT study, and three treatment groups: peanut 300, peanut 0 and placebo in the POISED study.

IMPACT participants were randomly assigned in a 2:1 allocation ratio, to receive peanut oral immunotherapy or placebo (oat flour) for 134 weeks (2000 mg peanut protein per day), followed by 26 weeks of avoidance (see **Figure S1**). There was an initial dose escalation (0.1 mg to 6 mg) of peanut flour or placebo, followed by maintenance of 2000 mg peanut protein or placebo daily until week 134. Treatment was discontinued and, after 26 weeks, a final evaluation took place. In POISED, all participants underwent build-up to and maintenance of 4000 mg PnOIT or placebo (oat flour) for 104 weeks. This was followed by treatment discontinuation (peanut 0 group), dose reduction to 300 mg peanut protein (peanut 300 group), and continued daily dosing of placebo group through to the end of the trial (see **Figure S1**).

In IMPACT, double-blind placebo-controlled food challenges (DBPCFCs) were conducted up to a cumulative dose of 500-mg peanut protein at study entry and only children who reacted at that or at a lesser dose were randomized. At the end of treatment (week 134) and after 26 weeks (week 160), the peanut DBPCFC reached up to a cumulative dose of 5000 mg peanut protein.

In POISED, the DBPCFC at study entry was performed up to a cumulative dose of 500 mg peanut protein and only participants who reacted at that or at a lesser dose were randomized. Subsequent food challenges starting at weeks 104, 117 and every 3 months thereafter were performed up to a cumulative dose of 4000 mg peanut protein (**Figure S1**). Genetic association was only evaluated through week 117, given decreasing sample sizes after.

In IMPACT, all subjects who were randomly assigned to treatment or placebo group comprised the intention-to-treat (ITT) sample, and ITT samples who adhered to maintenance dosing and avoidance per protocol and had an evaluable DBPCFC at weeks 134 and 160 comprised the per-protocol (PP) sample. In POISED, all randomized participants comprised the ITT sample and PP sample were defined as only individuals who passed a previous challenge and returned for the next challenge.

### Phenotypes included in genetic association analyses

In this study, we focused on testing the genetic association between the HLA-DQA1*01:02 allele and two phenotypes: (1) those who met or failed meeting the primary and secondary outcomes as defined in each study, and (2) the levels of psIgG4 and IgG4 to peanut components (Ara h 1, 2, 3, 6 in IMPACT, and Ara h 2 in POISED).

In IMPACT, the primary outcome is passing the DBPCFC of 5000 mg peanut flour challenge at 134 weeks (desensitization), and the secondary outcome is passing the 5000 mg DBPCFC 26 weeks after OIT discontinuation, i.e., week 160 week in the trial timeline (remission) (**Figure S1**). The levels of psIgG4 and IgG4 to Ara h 1, 2, 3, and 6 were measured at 5 timepoints: baseline (week 0), end of buildup period (week 30), during maintenance period (week 82 for psIgG4, and week 95 for IgG4 to peanut components), desensitization assessment (week 134) and remission assessment (week 160) timepoints. The levels of psIgG4 were measured using the ImmunoCAP 1000 system and IgG4 to peanut components (Ara h1, 2, 3, 6), were measured using the ImmunoCAP 250 system by Eurofins Viracor lab in Lees Summit. The lower limit of detection (LLOD) for psIgG4 was 0.075 ug/mL, and 0.0035 ug/mL for component specific IgG4. The levels of psIgG4 and IgG4 to peanut components were converted to ug/L and log_10_ transformed for genetic association testing.

In POISED, the primary outcome is passing the DBPCFC of 4000 mg at week 117. The secondary outcome is passing the 4000 mg DBPCFC to peanut at week 104, at the end of the maintenance phase. The levels of psIgG4 and IgG4 to peanut component Ara h 2 were measured at baseline (week 0), end of buildup period (week 52), desensitization assessment timepoint (week 104), and primary endpoint (week 117) using standardized methods in laboratory approved by Clinical Laboratory Improvement Amendments (Johns Hopkins University). The LLOD for both psIgG4 and IgG4 to Ara h 2 peanut component is 0.01 mg/L. We again log_10_ transformed the values of IgG4 phenotypes after converting the units to ug/L.

### Whole genome sequencing, quality control and imputation of HLA-DQA1*01:02 allele

Whole genome sequencing (WGS) to a depth of 30X coverage was performed using Illumina HiSeq X sequencer for N=126 IMPACT and N=120 POISED samples. The samples for WGS were prepared to the Illumina TruSeq Nano DNA library or TruSeq DNA PCR-free library preparation guides. Assembly of each individual genome was performed using the Isaac aligner (9). The DRAGEN Germline Small Variant Caller was used to call both SNVs and small indels, and to yield a genome variant file (gVCF) that includes variants along with quality metrics. We performed sample-based QC and dropped two samples because of sex inconsistencies from the POISED dataset. We also filtered variants with GQX < 30, DP < 7, SNP hard quality < 10.41, low depth DP <= 1, ploidy conflict, and variants not meeting thresholds for median base quality of alternate reads and likelihood.

We used the WGS data to impute HLA alleles using the HISAT-genotype software that utilizes HISAT2 (hierarchical indexing for spliced alignment of transcripts 2) alignment system to align DNA sequences using a graph Ferragina Manzini index (10). We had high quality (call rate) of imputation for HLA-DQA1*01:02 allele in both IMPACT (98.81%) and POISED (98.75%).

### Ancestry de-convolution of the IMPACT and POISED study participants

We used PC-AiR (11) to perform a principal components analysis (PCA) on WGS data for the detection of population structure. Unlike standard PCA, PC-AiR accounts for relatedness in the sample to provide accurate ancestry inference that is not confounded by family structure. The final association models included the first three principal components (PCs) for ancestry.

### Genetic association analyses

We performed the association testing between the HLA-DQA1 allele and phenotypes under a dominant model (i.e., carrier vs. non-carrier for each allele) in PLINK 1.9 (12). Logistic regression models were used for primary and secondary outcomes and linear models were used for the quantitative traits. In IMPACT, for the primary and secondary outcomes we tested the association with carrier status at the HLA-DQA1*01:02 allele including relevant baseline predictors from the original finding as covariates. Therefore, for the primary outcome, we included IgE to Ara h 6 at baseline, and for the secondary outcome we included baseline psIgE and age as covariates. For IgG4 phenotypes, age at baseline and sex were included as covariates. We corrected for ancestry with the inclusion of PCs derived on the genetic data in all the association models. The association models for primary and secondary outcomes, and IgG4 phenotypes at weeks 134 and 160 were performed in the PP sample.

The association tests in POISED, were also performed in PLINK with the same model specifications, except for covariate inclusion which were based on baseline predictors in original publication. In POISED, all the genetic association models were adjusted for age at baseline, sex and three genetic PCs as covariates. The association for IgG4 traits was tested in both PnOIT groups combined up to week 104, and separately in peanut 300 and peanut 0 groups at week 117. All hypothesis tests were two-sided.

## Results

### Study participant characteristics

A total of 126 participants of the original 146 participant study cohort from the IMPACT study, including N=85 participants in the PnOIT group, and N=41 in the placebo group are included. From the POISED study 118 participants from the initial study cohort of 120 participants are included in three treatment groups: peanut 300 (N=34), peanut 0 (N=59), and placebo (N=25) (**Table 1**). In IMPACT, the participants are similar in age, gender, baseline psIgE, and skin prick test at baseline but differences were noted in median cumulative tolerated dose at baseline between the treatment groups. In POISED, the participants are similar in age, gender, and baseline ascertainment criteria between treatment groups (**Table 1**). We note slight differences in ancestry groups for IMPACT that is likely reflective of small numbers of participants. For both studies, we adjusted for ancestry in the genetics analysis below.

**Table 1:** Demographics, baseline ascertainment criteria, clinical outcomes, and IgG4 levels of IMPACT and POISED genetic study participants.

IMPACT					POISED				
	Peanut OIT (N=85)		Placebo (N=41)	P-values		Peanut 300 (N=34)	Peanut 0 (N=59)	Placebo (N=25)	P-values
** Demographics**									
Age at enrollment (years), mean (SD)	3.04 (0.75)	3.05 (0.78)		NS	Age at enrollment (years), mean (SD)	14.7 (9.53)	13.1 (8.22)	15.4 (11.85)	NS
Male sex, (N)	68%, (58)	61% (25)		NS	Male sex, (N)	65%, (22)	64%, (38)	76%, (19)	NS
Female sex, (N)	32%, (27)	39% (16)			Female sex, (N)	35%, (12)	36%, (21)	24%, (6)	
***Ethnicity, (N)***					***Ethnicity, (N)***				
White/Caucasian	68%, (58)	63% (26)			White	65%, (22)	58%, (34)	72%, (18)	
Black or African American	0% (0)	7% (3)		4.15E-02	Black	3%, (1)	0%, (0)	4%, (1)	
Mixed race	15%, (13)	22% (9)			Multiracial	3%, (1)	15%, (9)	0%, (0)	NS
Asian	16%, (14)	7% (3)			Asian	29%, (10)	25%, (15)	24%, (6)	
					Native Hawaiian or Pacific Islander	0%, (0)	2%, (1)	0%, (0)	
**Baseline ascertainment criteria**									
SPTs (mm), median (IQR)	15 (12-18.5)	15 (11-20)		NS	SPTs (mm), median (IQR)	12.2 (8.5-15.5)	14.0 (8.5-18.2)	11.5 (9.5-14.5)	NS
psIgE (kU/L), median (IQR)	1.72 (1.43-2.28)	1.59 (1.30-2.29)		NS	psIgE (kU/L), median (IQR)	1.91 (1.11-2.25)	1.81(1.10-2.49)	1.88 (0.93-2.39)	NS
DBPCFCs, median (IQR)	75 (5-175)	25 (5-75)		7.68E-03	DBPCFCs, median (IQR)	25 (5-75)	25 (5-75)	25 (5-75)	NS
**Clinical Outcomes**									
***Primary Outcome, (N, Success/Failure/Missing)***					***Primary Outcome, (N, Success/Failure/Missing)***				
Desensitization	71%, (60/11/14)	2%, (1/28/12)		2.55E-06	Continued Desensitization	53%, (18/10/6)	NA	4%, (1/9/15)	5.00E-05
					Sustained Unresponsiveness	NA	34%, (20/30/9)	4%, (1/9/15)	4.70E-03
***Secondary Outcome, (N, Success/Failure/Missing)***					***Secondary Outcome, (N, Success/Failure/Missing)***				
Remission	21%, (18/45/22)	2%, (1/18/22)		1.40E-02	Desensitization	82%, (28/1/5)	NA	4%, (1/22/2)	6.92E-10
					Desensitization	NA	85%, (50/0/9)	4%, (1/22/2)	1.29E-12
***IgG4 phenotypes***					***IgG4 phenotypes***				
psIgG4 (ug/L) @ wk0	2.77 (2.45-3.22)	2.64 (2.18-3.08)		NS	psIgG4 (ug/L) @ wk0	3.04 (2.65-3.19)	2.68 (2.52-3.41)	2.90 (2.51-3.14)	NS
psIgG4 (ug/L) @ wk30	4.11 (3.77-4.44)	2.82 (2.51-3.12)		1.82E-14	psIgG4 (ug/L) @ wk52	4.14 (3.68-4.36)	4.10 (3.73-4.45)	2.80 (2.54-2.94)	6.32E-10
psIgG4 (ug/L) @ wk82/95	4.45 (4.12-4.92)	2.66 (2.46-3.07)		3.59E-18	psIgG4 (ug/L) @ wk104	4.33 (4.07-4.78)	4.29 (4.06-4.68)	2.66 (2.44-2.81)	7.57E-13
psIgG4 (ug/L) @ wk134	4.45 (4.13-4.45)	2.85 (2.45-3.40)		4.23E-14	psIgG4 (ug/L) @ wk117	4.17 (3.91-4.45)	3.97 (3.77-4.36)	2.71 (2.59-3.09)	2.31E-07
psIgG4 (ug/L) @ wk160	3.99 (3.50-4.15)	2.95 (2.71-3.59)		2.40E-05	Ara h 2 IgG4 (ug/L) @ wk0	2.22 (1.86-2.49)	2.18 (1.84-2.45)	2.04 (1.60-2.32)	NS
Ara h 1 IgG4 (ug/L) @ wk0	1.54 (1.54-2.20)	1.54 (1.54-2.23)		NS	Ara h 2 IgG4 (ug/L) @ wk52	3.95 (3.24-4.25)	4.01 (3.44-4.37)	1.70 (1.30-2.21)	3.59E-14
Ara h 1 IgG4 (ug/L) @ wk30	2.84 (2.15-3.42)	1.54 (1.54-2.26)		1.93E-10	Ara h 2 IgG4 (ug/L) @ wk104	4.29 (3.98-4.74)	4.22 (3.84-4.70)	2.11 (1.70-2.41)	2.65E-14
Ara h 1 IgG4 (ug/L) @ wk82/95	3.05 (2.35-3.69)	1.54 (1.54-2.33)		5.18E-11	Ara h 2 IgG4 (ug/L) @ wk117	4.06 (3.54-4.42)	3.84 (3.47-4.29)	2.23 (1.78-2.40)	5.01E-11
Ara h 1 IgG4 (ug/L) @ wk134	3.13 (2.41-3.70)	1.54 (1.54-2.40)		2.64E-10					
Ara h 1 IgG4 (ug/L) @ wk160	2.43 (1.54-2.92)	1.54 (1.54-2.30)		1.12E-03					
Ara h 2 IgG4 (ug/L) @ wk0	2.33 (2.04-2.60)	2.26 (1.54-2.53)		NS					
Ara h 2 IgG4 (ug/L) @ wk30	3.96 (3.61-4.29)	2.26 (1.54-2.62)		7.63E-26					
Ara h 2 IgG4 (ug/L) @ wk82/95	4.46 (4.13-4.78)	2.28 (1.54-2.50)		7.50E-28					
Ara h 2 IgG4 (ug/L) @ wk134	4.44 (4.09-4.75)	2.20 (1.54-2.49)		6.11E-25					
Ara h 2 IgG4 (ug/L) @ wk160	3.80 (3.49-4.03)	2.28 (1.77-2.59)		7.41E-10					
Ara h 3 IgG4 (ug/L) @ wk0	2.18 (1.54-2.53)	2.20 (1.54-2.65)		NS					
Ara h 3 IgG4 (ug/L) @ wk30	3.18 (2.74-3.62)	2.11 (1.54-2.70)		5.13E-07					
Ara h 3 IgG4 (ug/L) @ wk82/95	3.60 (3.21-4.00)	2.28 (1.54-2.80)		1.47E-09					
Ara h 3 IgG4 (ug/L) @ wk134	3.63 (3.21-4.02)	2.18 (1.54-2.52)		3.43E-09					
Ara h 3 IgG4 (ug/L) @ wk160	3.15 (2.71-3.63)	2.30 (1.54-2.97)		6.00E-04					
Ara h 6 IgG4 (ug/L) @ wk0	2.18 (1.54-2.43)	2.20 (1.54-2.51)		NS					
Ara h 6 IgG4 (ug/L) @ wk30	3.97 (3.62-4.36)	2.28 (1.54-2.57)		1.58E-24					
Ara h 6 IgG4 (ug/L) @ wk82/95	4.43 (4.06-4.80)	2.18 (1.77-2.62)		2.19E-26					
Ara h 6 IgG4 (ug/L) @ wk134	4.35 (4.02-4.72)	2.11 (1.54-2.58)		2.78E-23					
Ara h 6 IgG4 (ug/L) @ wk160	3.66 (3.27-4.02)	2.30 (1.54-2.94)		7.82E-08					

In the IMPACT study, statistical significance for continuous data was tested using t-test and for dichotomous variables was tested using Pearson’s Chi-squared test and Fisher exact test.

In the POISED study, statistical significance for continuous data was tested using ANOVA, and for dichotomous variables was tested using the Pearson’s Chi-squared test and Fisher exact test.

In IMPACT, DBPCFCs to 500 mg peanut protein and in POISED, DBPCFCs to 4000 mg peanut protein were performed and highest cummulative tolerated dose (mg) is presented.

The results for primary and secondary outcomes are slightly different from the parent publications because of different Ns in this genetics study.

NS, Not significant.

In IMPACT, a greater proportion of participants in the PnOIT group achieve desensitization (upon completion of therapy) and disease remission (assessed after 26 weeks of therapy) as compared to the placebo group. Both desensitization and remission were evaluated on the basis of a double-blind placebo-controlled food challenge (DBPCFC) at week 134 and 160, respectively (see **Methods**). The levels of psIgG4 and IgG4 to peanut components Ara h 1, 2, 3, and 6 are significantly different between the groups (**Table 1**). In POISED, greater proportions attain desensitization (peanut 300 group) and the SU (peanut 0 group) on the basis of passing a DBPCFC compared to the placebo group at week 117. Similarly, desensitization at week 104 was significantly higher in both OIT groups than the placebo group. The levels of psIgG4 and IgG4 to Ara h 2 are also significantly different between the treatment groups (**Table 1**).

### Association of the HLA-DQA1*01:02 allele with psIgG4 and IgG4 to peanut components in IMPACT and POISED

The allele frequency of HLA-DQA1*01:02 allele was 24% in IMPACT and 20% in POISED; 47% of participants in IMPACT and 36% in POISED were found to be carriers (i.e. had one or two copies of the allele). To follow up on our finding of gene environment interaction between the HLA allele and IgG4 in the consumption arm of LEAP, we tested for the association of HLA-DQA1*01:02 with levels of psIgG4 and IgG4 to four individual peanut components (Ara h 1, h 2, h 3 and h 6) in IMPACT, and psIgG4 and IgG4 to Ara h 2 in POISED (**Table 2**). In IMPACT, the association was tested at weeks 0, 30, 82/95 (see methods), 134 and 160. HLA-DQA1*01:02 is significantly associated with psIgG4 at week 30 (p=9x10^-3^) in the PnOIT group with higher psIgG4 observed in the carriers of the HLA-DQA1*01:02 allele. The association diminishes at later timepoints (p>0.05, **Table 2**; **Figure S2**). The allele also shows significant association with IgG4 to peanut components Ara h 2 and Ara h 6 at week 30 (Ara h 2, p=5x10^-3^ and Ara h 6, p=1x10^-3^) and with Ara h 6 at week 95 (p=1.4x10^-2^) in the PnOIT group, but no associations were observed after PnOIT discontinuation (weeks 134-160). Consistently, in the PnOIT group, carriers of the HLA-DQA1*01:02 allele had higher component specific IgG4 than non-carriers. On the other hand, in the placebo group no associations are noted at any time points (**Table 2**). In POISED, the associations of HLA allele with psIgG4 and IgG4 to Ara h2 were tested at four timepoints: weeks 0, 52 104 and 117 in both PnOIT and placebo groups. No associations are noted between HLA-DQA1*01:02 and IgG4 at any timepoint in any treatment group (**Table 2**).

**Table 2:** Association between HLA-DQA1*01:02 and IgG4 in IMPACT and POISED. Table shows the effect size for the log transformed phenotype (Est) and p-values from the linear regression models, and results with p<0.05 are bolded.

IMPACT										
Phenotypes	Week 0		Week 30		Week 82/95		Week 134*		Week 160*	
	Est	P-value	Est	P-value	Est	P-value	Est	P-value	Est	P-value
PnOIT	(N=85)		(N=79)		(N=73)		(N=71)		(N=63)	
**psIgG4**	0.199	0.135	**0.364**	**0.009**	0.157	0.309	0.208	0.239	0.248	0.125
**Ara h 1 IgG4**	-0.179	0.086	-0.092	0.612	-0.117	0.603	-0.098	0.663	-0.034	0.854
**Ara h 2 IgG4**	0.165	0.116	**0.426**	**0.005**	0.358	0.055	0.235	0.212	0.348	0.064
**Ara h 3 IgG4**	0.164	0.334	0.048	0.767	0.021	0.897	0.036	0.836	0.105	0.545
**Ara h 6 IgG4**	0.172	0.111	**0.506**	**0.001**	**0.423**	**0.014**	0.306	0.103	0.373	0.055
**Placebo**	(N=41)		(N=37)		(N=32)		(N=29)		(N=19)	
**psIgG4**	0.111	0.673	0.106	0.693	0.059	0.838	0.094	0.797	0.288	0.545
**Ara h 1 IgG4**	-0.023	0.876	-0.168	0.377	-0.155	0.453	-0.189	0.475	-0.357	0.228
**Ara h 2 IgG4**	0.309	0.101	0.271	0.181	0.421	0.075	0.434	0.128	0.661	0.108
**Ara h 3 IgG4**	0.459	0.125	0.351	0.282	0.319	0.367	0.173	0.690	0.487	0.304
**Ara h 6 IgG4**	0.306	0.131	0.224	0.316	0.189	0.452	0.003	0.993	0.238	0.661
POISED										
Phenotypes	Week 0		Week 52		Week 104		Week 117**			
	Est	P-value	Est	P-value	Est	P-value	Est		P-value	
**Peanut 300 + Peanut 0**	(N=93)		(N=81)		(N=79)		(Peanut 300, N=28; Peanut 0, N=40)			
**psIgG4**	0.055	0.690	0.025	0.880	0.064	0.700	0.205/0.025		0.646/0.647	
**Ara h 2 IgG4**	0.051	0.700	0.081	0.700	0.123	0.550	0.473/0.011		0.381/0.963	
**Placebo**	(N=25)		(N=23)		(N=23)		(N=16)			
**psIgG4**	0.265	0.421	0.654	0.107	0.727	0.060	0.322		0.325	
**Ara h 2 IgG4**	-0.019	0.941	0.540	0.313	0.708	0.158	0.249		0.592	

*In IMPACT, association analyses at weeks 134 and 160 in both PnOIT and Placebo groups are limited to per protocol participants.

**In POISED, association analyses at week 117 was stratified by Peanut 300/Peanut 0 due to difference in peanut exposure status after week 104 between the two groups.

### Association of the HLA-DQA1*01:02 allele with primary and secondary outcomes in PnOIT groups of IMPACT and POISED

Association with the HLA-DQA1*01:02 allele was tested with desensitization and remission outcomes in the PnOIT group of IMPACT. We observed that – albeit not statistically significant – a higher proportion of carriers (C) of the HLA allele, compared to non-carriers (NC), showed desensitization (C=93% vs. NC=78%, OR=5.74, p=0.06) and remission (C=35% vs. NC=22%, OR=1.26, p=0.80) (**Table 3**). While sample sizes are too small to perform statistical tests for association, we did look at a further stratification of the IMPACT study participants by age at entry (12-23.9 months, 24-35.9 months, and 36-47.9 months). Similar to the prior findings (5), in this group with available genetic data, a higher rate of remission was noted in the youngest participants on pnOIT: 66.6%, 35.0% and 18.9% in the 12-23.9 (N=6), 24-35.9 (N=20) and 36-47.9 (N=37) groups, respectively. This was even more notable in those participants carrying the HLA-DQA1*01:02 where remission was 80%, 40% and 18.75% in the 12-23.9 (N=5), 24-35.9 (N=5) and 36-47.9 (N=16) groups, respectively.

**Table 3:** Association between HLA-DQA1*01:02 and primary and secondary outcomes in IMPACT and POISED. Table shows the percentage of carriers and non-carriers with each outcome, the odds ratio (OR) and p-value.

IMPACT		Carriers N(%)	Non-Carriers N(%)	OR	P-value
**Primary Outcome**	Desensitized	28 (93%)	31 (78%)	5.74	0.06
**PnOIT group**	Non-desensitized	2 (7%)	9 (23%)		
**Secondary Outcome**	Remission	9 (35%)	8 (22%)	1.26	0.80
**PnOIT group**	No-remission	17 (65%)	28 (78%)		
**POISED**		**Carriers N(%)**	**Non-Carriers N(%)**	**OR**	**P-value**
**Primary Outcome**	SU	12 (52%)	8 (31%)	2.32	0.19
**Peanut 0 group**	Not-SU	11 (48%)	18 (69%)		
**Primary Outcome** **Peanut 300 group**	Continuous desensitization	4 (80%)	14 (61%)	1.28	0.86
	Not-Continuous desensitization	1 (20%)	9 (39%)		

SU, sustained unresponsiveness.

In POISED, the desensitization outcome was not assessed as the majority of participants were successfully desensitized. The association was tested with continued desensitization in the peanut 300 group, and with SU in the peanut 0 group (**Table 3**). We found that HLA-DQA1*01:02 carriers (C) were more frequently desensitized to 4,000 mg than non-carriers (NC) in the peanut 300 group (C=80% vs. NC=61%; OR=1.28, p=0.86). Similar patterns were noted for SU in the peanut 0 group (C=52% vs. NC=31%; OR=2.32, p=0.19). While none of these associations were statistically significant, there was consistency in the observation of better outcomes in carriers in all instances of PnOIT.

## Discussion

HLA-restricted recognition of specific antigens is a fundamental genetically determined checkpoint permissive for immune responses. Oral immunotherapy to food allergens is an example of controlled antigen exposure with therapeutic potential, and the relationship between HLA-DQA1*01:02 and peanut allergy provides a framework for improving our understanding of allergic sensitization and tolerance. In the LEAP clinical trial, where oral peanut exposure was initiated early (age <11months) in children at high risk for peanut allergy, the elevation of psIgG4 was significantly associated with the combination of this specific genotype and consumption of peanut. The observed interaction is consistent with the concept that immune responses in children with specific HLA class II genotypes preferentially recognize specific allergens during oral antigen exposure in therapeutic allergen immunotherapy. Since IgG4 serves as a blocking antibody limiting the interaction of peanut allergen with IgE on the surface of mast cells and basophils (13), our findings in LEAP would imply that individuals with the HLA-DQA1*01:02 allele present particular peanut allergenic peptides (i.e., Ara h 2) for immune recognition that invokes protective mechanisms when introduced to the diet prior to the development of peanut allergy. In addition to HLA-DQA1*01:02 allele, we also observed the association between the psIgG4 and an intergenic SNP rs17612852 in LEAP, but noted strong linkage disequilibrium (LD) and the inability to tease apart the proportion of variance in psIgG4 explained by each variant separately. Here too, we had high LD between the allele and previously reported SNP (e.g., in IMPACT the LD is: D’=1, r2 = 0.62), and even smaller sample sizes (e.g., N=85 in IMPACT PnOIT group). Therefore, this follow-up study in IMPACT and POISED trials is limited to HLA-DQA1*01:02 allele only.

Following the LEAP trial, two subsequent trials- POISED and IMPACT, where peanut oral immunotherapy was administered in a randomized design to individuals with established peanut allergy provide an opportunity to test and extend several observations that support our hypothesized mechanism. In POISED and IMPACT, the study sample sizes were much smaller than LEAP, limiting our ability to reach statistical significance in any single group. However, in all instances, the primary and secondary outcomes analyses point towards better therapeutic outcomes following PnOIT for carriers of the HLA-DQA1*01:02 allele. The stronger evidence was noted in young children in IMPACT (age 12-<48 months at trial entry) where carriers were more frequently desensitized and more frequently had peanut allergy remission than non-carriers with an OR=5.74 and OR=1.26, respectively; and rates of remission were highest (80%) in the youngest participants (age <24 months) that were carriers of the allele. While the IMPACT trial reported that participants younger participants achieved greater remission, unfortunately, the samples sizes within IMPACT limit our ability to test specifically for age-related effects related to genetics. In IMPACT, we also found associations of the HLA-DQA1*01:02 allele with increased psIgG4 that was similar to the associations noted in LEAP; higher psIgG4 was noted in carriers, and importantly this was specific for Ara h 2 and Ara h 6. While in LEAP, we only tested Ara h 2, here the strength of association with Ara h 6 in addition to Ara h 2 further validates the specificity of the role of HLA; Ara h 2 and 6 are storage proteins of the 2S albumin and potent peanut allergens and are known to have similar allergenic activity and have higher homology (14, 15). Better therapeutic outcomes from peanut OIT were also noted in the carriers of the HLA-DQA1*01:02 allele in POISED for both sustained unresponsiveness after PnOIT discontinuation (SU) and continued desensitization despite PnOIT reduction (OR = 2.32 and 1.28, respectively) although here too, both groups were too small to achieve significance. Notably, we do not see any association with psIgG4 in the peanut OIT arms of POISED which enrolled a substantially older population (age in years: 11 (8–17) for peanut 300, and 10 (9–13) for peanut 0) of peanut allergic individuals.

These findings illustrate a nuanced context-dependent contribution of HLA-restricted allergen recognition to peanut allergy and response to therapy that may be age dependent. While the specific mechanism of the HLA effects by age are not established at this time, the observation of age dependent disease risk on the basis of HLA genetics has previously been noted across a variety of immune-related diseases (16–23). Here, the very same HLA DQA1 allele is associated with peanut allergy in population cross-sectional studies and with protection from peanut allergy in the LEAP peanut oral prevention trial, where the age at intervention was <11 months, consistent with the concept that mode of exposure distinguishes between sensitization and tolerance outcomes. This effect correlated with the induction of peanut-specific IgG4, suggesting a direct therapeutic mechanism associated with clinical benefit. In the IMPACT trial, higher levels of psIgG4 in allergic children between 12 and 48 months of age also was associated with the same allele, with remarkably similar effects as noted in LEAP. There were also trends noted for favorable outcomes in these younger participants from IMPACT. And in POISED, where the age of enrollment was the highest of the three clinical trials, 7-55 years, although there was a similar trend towards favorable outcomes, no association with psIgG4 was found. These studies suggest a gene-exposure continuum in which specific HLA-dependent antigen presentation events lead to subsequent sensitization or protective immunologic responses that are sensitive to context: Oral route of exposure and early age are indicative of a favorable therapeutic outcome in HLA-DQA1*01:02 individuals most evident in non-allergic infants, somewhat less effective in allergic children, and substantially less apparent in allergic adolescents and adults. This paradigm supports the concept that early age intervention with oral immunotherapy, particularly in genetically selected individuals, can be a potent strategy for interdicting the allergic diathesis by redirecting immune responses towards favorable outcomes. Further studies are necessary to better understand the mechanisms through which genetic risk appears to be modifiable by oral exposure only in window of the first few years of life.

## Data availability statement

The data presented in the study are deposited in the dbGaP repository, accession numbers phs003071.v1.p1 and phs003109.v1.p1.

## Ethics statement

The studies involving human participants were reviewed and approved by FDA investigational new drug application and monitored by a National Institutes of Health (NIH)–National Institute of Allergy and Infectious Diseases (NIAID) Data and Safety Monitoring Board for IMPACT. And by the Division of Allergy, Immunology, and Transplantation (DAIT) and the National Institute of Allergy and Infectious Diseases (NIAID) Allergy and Asthma Data Safety Management Board, the DAIT/NIAID Clinical Review Committee, the Stanford Institutional Review Board, and the US Food and Drug Administration (FDA) for POISED. The IMPACT trial is registered on ClinicalTrials.gov as NCT03345160 and POISED as NCT02103270. Written informed consent to participate in this study was provided by the participants’ legal guardian/next of kin.

## Author contributions

KK, GS, HL, and JP performed the analysis. KK, GS, RC, SS, MM, HL, JP, KN and SJ generated the data. KK, MH, RC, SS, MM, GT, AT, GL, KN, SJ, GN and RM were involved in the design of the study. KK, GS, MH, HB, RC, SS, MM, HL, JP, GT, IR, AT, GL, KN, SJ, GN and RM wrote and edited the manuscript. All authors contributed to the article and approved the submitted version.

## Funding

This research was performed as a project of the Immune Tolerance Network, an international clinical research consortium headquartered at the Benaroya Research Institute and supported by the National Institute of Allergy and Infectious Diseases (NIAID) of the National Institutes of Health (NIH) under award no. UM1AI109565. IMPACT trial was funded through NIH-NIAID under Award Number UM1AI109565 and UM2AI117870. Other sources of support include the National Center for Research Resources supported Clinical Translational Science Awards and Clinical Research Centers TR003107 (University of Arkansas for Medical Sciences), TR001111 (University of North Carolina), TR003142 (Stanford University), TR000067 (Mount Sinai University), and TR000424 (Johns Hopkins University School of Medicine). POISED trial was supported by the NIAID grant AADCRC U19AI104209, the Sean N Parker Center for Allergy and Asthma Research at Stanford University, the Myra Reinhard Family Foundation, Food Allergy Research & Education Center of Excellence, End Allergies Together, TripAdvisor Charitable Foundation, and Crown Family Philanthropies.

## Acknowledgments

We thank Dr Daniel Rotrosen from the National Institute of Allergy and Infectious Diseases for his thoughtful review of the manuscript. The content is solely the responsibility of the authors and does not necessarily represent the official views of the National Institutes of Health. We thank the nurses, dietitians, study coordinators, laboratory staff, and other research staff at each institution and the NIAID Division of Allergy, Immunology and Transplantation’s Statistical and Clinical Coordinating Center at Rho involved in the IMPACT study. For POISED trial, the study drug was manufactured through the Stanford good manufacturing practice (GMP) facility as per good clinical practice and GMP guidelines. All quality assurance and quality control were done as per US Food and Drug Administration GMP guidelines. We thank Dana Tupa, Sayantani Sindher, Vanitha Sampath, the nurses, dieticians, study coordinators, other staff involved in the POISED study. We are grateful to all the IMPACT and POISED participants and their families who took part in the study.

## Conflict of interest

GL reports holding stock and stock options in DBV Technologies. HB has provided statistical consulting to DBV Technologies (paid to Benaroya Research Institute).

The remaining authors declare that the research was conducted in the absence of any commercial or financial relationships that could be construed as a potential conflict of interest.

## Publisher’s note

All claims expressed in this article are solely those of the authors and do not necessarily represent those of their affiliated organizations, or those of the publisher, the editors and the reviewers. Any product that may be evaluated in this article, or claim that may be made by its manufacturer, is not guaranteed or endorsed by the publisher.

## Author disclaimer

Dr. Togias’ co-authorship of this article does not constitute endorsement by the National Institute of Allergy and Infectious Diseases, the National Institutes of Health or any other Agency of the United States Government.
