# Supplementary material for: HLA-associated outcomes in peanut oral immunotherapy trials identify mechanistic and clinical determinants of therapeutic success
Source: Front Immunol. 2022 Nov 18;13:941839. doi: 10.3389/fimmu.2022.941839 (PMC9717393; doi:10.3389/fimmu.2022.941839)

**Figure S1: Overview of the IMPACT and POISED clinical trials.** The study design and key features of IMPACT (5) and POISED (6) clinical trials adapted from original publications are shown here. A summary of treatment groups, time points at which double blinded placebo controlled food challenges (DBPCFCs) were performed, and timepoints at which levels of psIgG4 were measured in both studies are shown below in the study schematics.

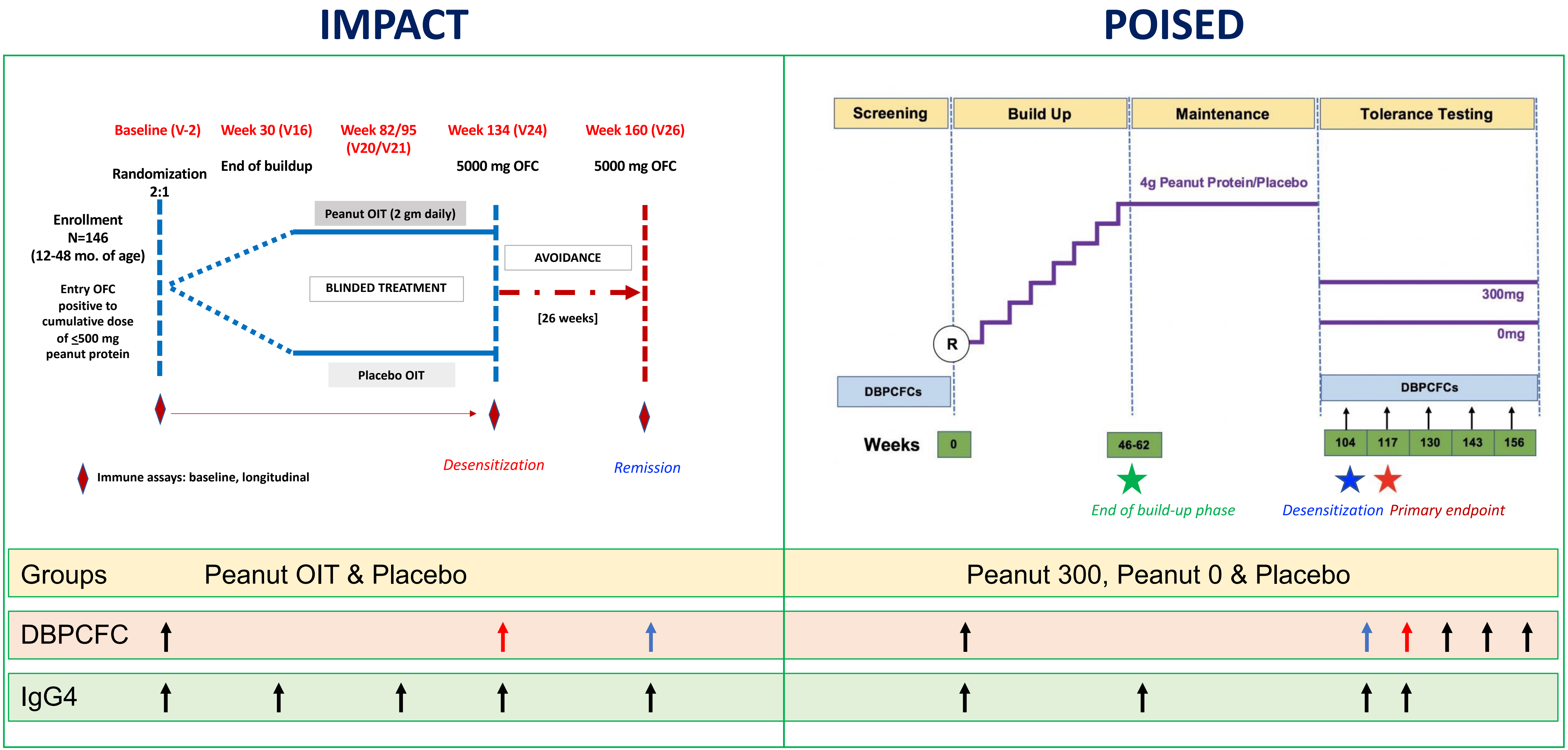

Supplement: Supplementary file 1 [file Image_1.pdf]
