# Supplementary material for: HLA-associated outcomes in peanut oral immunotherapy trials identify mechanistic and clinical determinants of therapeutic success
Source: Front Immunol. 2022 Nov 18;13:941839. doi: 10.3389/fimmu.2022.941839 (PMC9717393; doi:10.3389/fimmu.2022.941839)

Figure S2: Distribution of psIgG4, and component specific IgG4 to Ara h2 and Ara h6 in the IMPACT participants by intervention (Placebo and PnOIT) and HLA~DQA1*01:02 carrier status (NC = non-carrier, C = carrier). Significance associations from Table 2 are indicated in red.

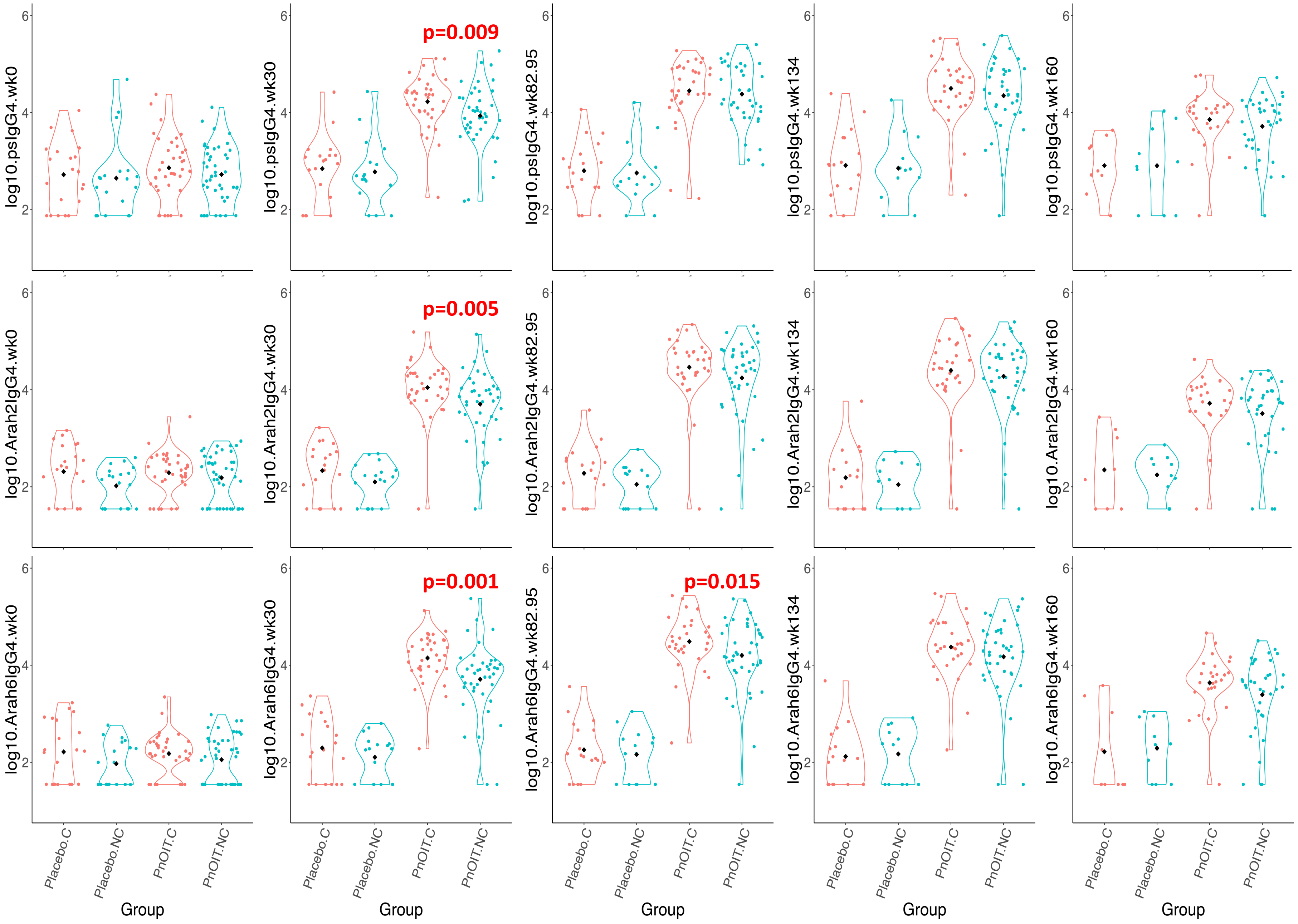

Supplement: Supplementary file 2 [file Image_2.pdf]
